# Pathological features of transplanted tumor established by CD133 positive TJ905 glioblastoma stem-like cells

**DOI:** 10.1186/s12935-015-0208-y

**Published:** 2015-06-13

**Authors:** Feng Jin, Ran Zhang, Song Feng, Chuan-Tao Yuan, Ren-Ya Zhang, Guang-Kui Han, Gen-Hua Li, Xi-Zhen Yu, Yang Liu, Ling-Sheng Kong, Shu-Ling Zhang, Lei Zhao

**Affiliations:** Department of Neurosurgery, Affiliated Hospital of Jining Medical University, and Shangdong Provincial Key Laboratory of Stem Cells and Neuro-oncology, Jining, Shandong 272029 PR China; Department of Pathology, Affiliated Hospital of Jining Medical University, Jining, Shandong 272029 PR China; Department of Infectious Diseases, Union Hospital, Tongji Medical College, Huazhong University of Science and Technology, Wuhan, 430022 PR China

**Keywords:** CD133, Glioblastoma, Cancer stem-like cell, Animal model, Transplanted tumor

## Abstract

**Background:**

This study is to explore the pathological features of transplanted tumor established by CD133 positive TJ905 glioblastoma stem-like cells.

**Methods:**

CD133 positive TJ905 glioma cells were separated by immunomagnetic beads to isolate glioma stem-like cells. TJ905 cells and stem-like cells were inoculated subcutaneously into the mice to establish model of transplanted tumor, respectively. Mice growing condition and behavior were observed. HE staining assay, immunohistochemical assay for GFAP, Ki-67 and Olig-2, and CD34 marked microvascular density (MVD) test were performed.

**Results:**

The growing condition and behavior of mice in TJ905 stem cell group was more exaggerated and the models showed stronger malignant features pathologically than that in TJ905 cell group. Glial fibrillary acidic protein (GFAP) in TJ905 cell and stem-like cell group showed the transplanted tumor originated from astrocytes. Expression of Ki-67 and oligodendrocyte transcription factor-2 (Olig-2) in TJ905 stem cells was higher notably and CD34 expression in stem cell group was significantly higher than that in the other two groups.

**Conclusions:**

Pathological features of transplanted tumor established by CD133 positive glioblastoma stem-like cells show more malignant. Use of TJ905 stem cells to establish transplanted tumor model in nude mice is excellent for glioma research.

## Introduction

Glioblastoma Multiforme (GBM) is the most common adult primary malignant brain tumors. Despite only 1.5 % in all tumors, GBM is the most lethal tumor [1]. Although the best option, standard surgical resection with radiotherapy and chemotherapy, is performed, the median survival time of patients is 12 to 18 months and most patients died within 2 years after diagnosis [2]. Currently the strategy to cure glioblastoma is still facing great challenges, especially drug resistance and tumor recurrence after treatment [3]. It is necessary to find new solutions for treating this malignance by studying glioblastoma at molecular and cellular levels.

Current evidence indicates that a subpopulation of cancer cells, named cancer stem cells (CSCs) or tumor-initiating cells which are responsible for the initiation, growth, metastasis, therapy resistance and recurrence of cancers, share core regulatory pathways with normal stem cells but rely on distinct reprogrammed pathways to maintain stemness and to contribute to the progression of cancers [4]. In recent years, many studies found that there are a small group of cancer stem-like cells in glioblastoma multiforme and they have features similar to normal neural stem cells, such as self-renewal and differentiation capacity. The group of cells might be an important cause for recurrence of GBM and resistance to chemotherapy [5]. Those glioma stem cells show characteristics including higher tumorigenic potential, low multiplication rate, high expression of CD133 and specific genes of neural stem cell [6]. As many mutations in the genome of most common glioma cell lines, it is postulated that the cancer stem-like cells can establish a more representative GBM model like the primary tumor in genotype [7, 8].

Thereby, we used CD133+ GBM cancer stem-like cells to establish animal models, in order to provide a new experimental platform with more similarity to the characteristics of primary cancer. In our study, glioma stem cells were injected into nude mice subcutaneously for setting up implanting tumor model. Tumor growth condition, behavioral changes, histopathology and immunohistochemical staining was observed in the model to improve the further understanding of glioblastoma on cancer etiology, gene therapy and pharmacodynamics.

## Materials and methods

### Chemicals and reagents

Dulbecco’s Modified Eagle’s Medium/Nutrient Mixture F-12 Ham’s (DMEM/F12) with high glucose medium was purchased from HyClone (Logan, UT). Fetal bovine serum (FBS), trypsin, B-27 (1×) Serum-Free Supplements was from Gibico (Grand Island, NY). Epidermal growth factor (EGF), basic fibroblast growth factor (bFGF), leukemia inhibitory factor (LIF) was obtained from Peprotech (Rocky Hill, NJ). CD133 magnetic activated cell sorting (MACS) kit was taken from Miltenyi Biotec GmbH (Bergisch Gladbach, Germany). The hematoxylin and eosin (HE) staining kit was provided from Boster Biological Technology, Ltd (Wuhan, China). SP immunohistochemistry kit was offered from Zhongshan Goldenbridge Biotechnology Co., Ltd (Beijing, China).

### Cell culture of TJ905 cells

The TJ905 cell line was established and characterized by Tianjin Neurological Institute laboratory of Neuro-oncology [9]. TJ905 cell line was purchased from China center for typical culture collection (CCTCC). TJ905 cells were cultured in DMEM/F12 medium with 10 % FBS, 100U/ml penicillin and 100ug/ml streptomycin. The culture was in a 37 °C, 5 % CO_2_ saturated humidity incubator.

### Isolation and culture of TJ905 stem cells

TJ905 cell line was cultured with medium for neural stem cell including DMEM/F12,20 ng/ml EGF, 20 ng/ml bFGF, 10 ng/ml LIF and B27(1X). The culture was in a 37 °C, 5 % CO_2_ saturated humidity incubator with renewing medium for every 3–4 days. After a large group of spheres of stem cells appeared, the spheres were collected and MACS was used to isolate CD133+ cells [10].

### Animals

Thirty-five male 5-week old nude mice with 10–15 g weight were purchased from Hubei Provincial Experimental Animal Center. All animal study protocols were approved by internationally accepted principles and the Guidelines for the Care and Use of Laboratory Animals of Huazhong University of Science and Technology [11].

### Animal grouping

The animals were divided into 4 groups, TJ905 cell group, TJ905 stem cell group, TJ905 CD133- group and normal control group. The former three groups were prepared by 10 mice in each group and the latter was given 5 mice for experiment. The mice in TJ905 cell group were injected with 1.0 × 10^6^ cells and in TJ905 stem cell group the mice were given 5000 stem cell spheres, while the mice in normal control were treated by D-hanks solution with equal volume [8].

### Cells inoculated subcutaneously in nude mice

After weighing, 3 % iodine and 75 % alcohol was used to sterilize the abdomen and right thigh puncture site. The nude mice were injected peritoneally with 10 % chloralhydrate 0.3 ml per 100 g for anesthesia. Then 100 μl 10^6^ cell suspension was inoculated at the right thigh subcutaneously by microlitre syringe with 1 μl/min speed slowly. Prior to withdrawing, the needle stayed for 5 min in order for deposition of the cells. The whole procedure was performed in a sterile environment and no use of antibiotics.

### Observation on mice growing condition and behavior

The inoculated mice were fed and observed in Experimental Animal Center of Tongji Medical College. The weight, skin cleanness, drinking and eating, activity and semiplegia of the mice were observed.

### Measurement of transplanted tumor size

At each 3 days after 7th day of inoculation, the subcutaneous neoplasms of nude mice in 2 model groups were measured by caliper rule, respectively. The tumor diameter was measured and tumor size was calculated. Tumor sizes in the two groups were compared.

### Sample collection

Twenty-one day after the inoculation of cells or spheres, each mouse in first three groups was injected 10 % chloralhydrate for hyperanesthesia. 5 mice in TJ905 cell and stem cell group were injected 10 % chloralhydrate for hyperanesthesia. The mice were executed and tumor tissue was taken out. The tissue was washed with cold normal saline and conserved in liquid nitrogen tank quickly for test. The rest tissue was fixed quickly with 4 % paraformaldehyde for 2–4 h and then was placed in 30 % sucrose solution overnight. At last the tissue was embedded with paraffin. The heart, liver, lung, spleen, kidneys, and spinal cord were also taken for examination to confirm whether metastasis occurred. The samples in normal control group were taken at the end of experiment.

### HE staining assay

Paraffin-embedded tissues were cut to 4 μm slices and were deparaffinate in dimethylbenzene for 5–10 min. Then the tissues were put into 100 %, 95 %, 85 % and 70 % alcohol for 2–5 min in turn and finally washed by distilled water and immersed into staining solution. After hematoxylin staining for 5–15 min, the excess stain solution on the slides was washed, and colorseperation with 0.5–1 % hydrochloride alcohol (made by 75 % alcohol) was performed for about 10 s. Following washed with running water for 15–30 min, the tissues were stained by 0.1 ~ 0.5 % eosin for 1–5 min. Prior to hyalinized with dimethylbenzene twice for about 10 min in total, the tissues were dehydrated with 75 %, 85 %, 95 %, 100 % alcohol for 2–3 min in turn. At last slipp was droped by neutral gum and covered by slide. As a result, nucleuses were blue and cytoplasm, collagen fibers showed different red or pink.

### Immunohistochemical assay on GFAP, Ki-67 and Olig-2

Streptavdin-peroxidase-biotin (SP) method was used for immunohistochemistry. The slides were deparaffinated conventionally and were immersed with 3 % H2O2 for 10 min to block endogenous peroxidase. After antigen retrieval by microwave, new-born calf serum was added for blocking for 10 min, and then first antibody (GFAP 1:50, Ki-67 and Olig-2 primary concentration) was added to incubate overnight (4 °C) and secondary antibodies to incubate for 20 min at room temperature. Then streptavidin-biotin-peroxidase solution was used to incubate for 30 min and 3,3′-diaminobenzidine (DAB) was added to chlorate for 15 min. Followed by hematoxylin staining, dehydration and hyalinization, the slip was covered. In the negative control, first antibody was replaced by phosphate buffered saline (PBS). As a result, glial fibrillary acidic protein (GFAP), Ki-67 and oligodendrocyte transcription factor-2 (Olig-2) positive staining located in the cytoplasm and cell protrusion and showed brown. The slices were observed in high power microscopy and positive cell counting in each 50 visual fields were averaged.

### CD34 marked microvascular density (MVD) test

CD34 expresses in vascular endothelial cells, tumor cytoplasm or membrane and is used to a specific marker of vascular endothelial cell. Immunohistochemical staining makes it show a distribution of brown or light brown solid bud-like or cord-like blood vessels. For observation, the fields at which tumor micrangium count was most at low magnification vision were selected, and then the vascular endothelial cells or cell group which showed brown or sepia at high magnification (×200 times) were counted. One should be counted as independent micrangium on condition of obvious distinction from neighbor micrangium and tumorous cell. 5 counts of micrangium of each slide at high magnification vision were recorded, and the average was taken as the MVD.

### Statistical analysis

Measurement data shows as mean ± SD. Group comparison was compared with one-way ANOVA analysis. Significance was considered when *P* < 0.05. All the data were analyzed with SPSS 12.0.

## Results

### Mice growing condition

**Fig. 1:**
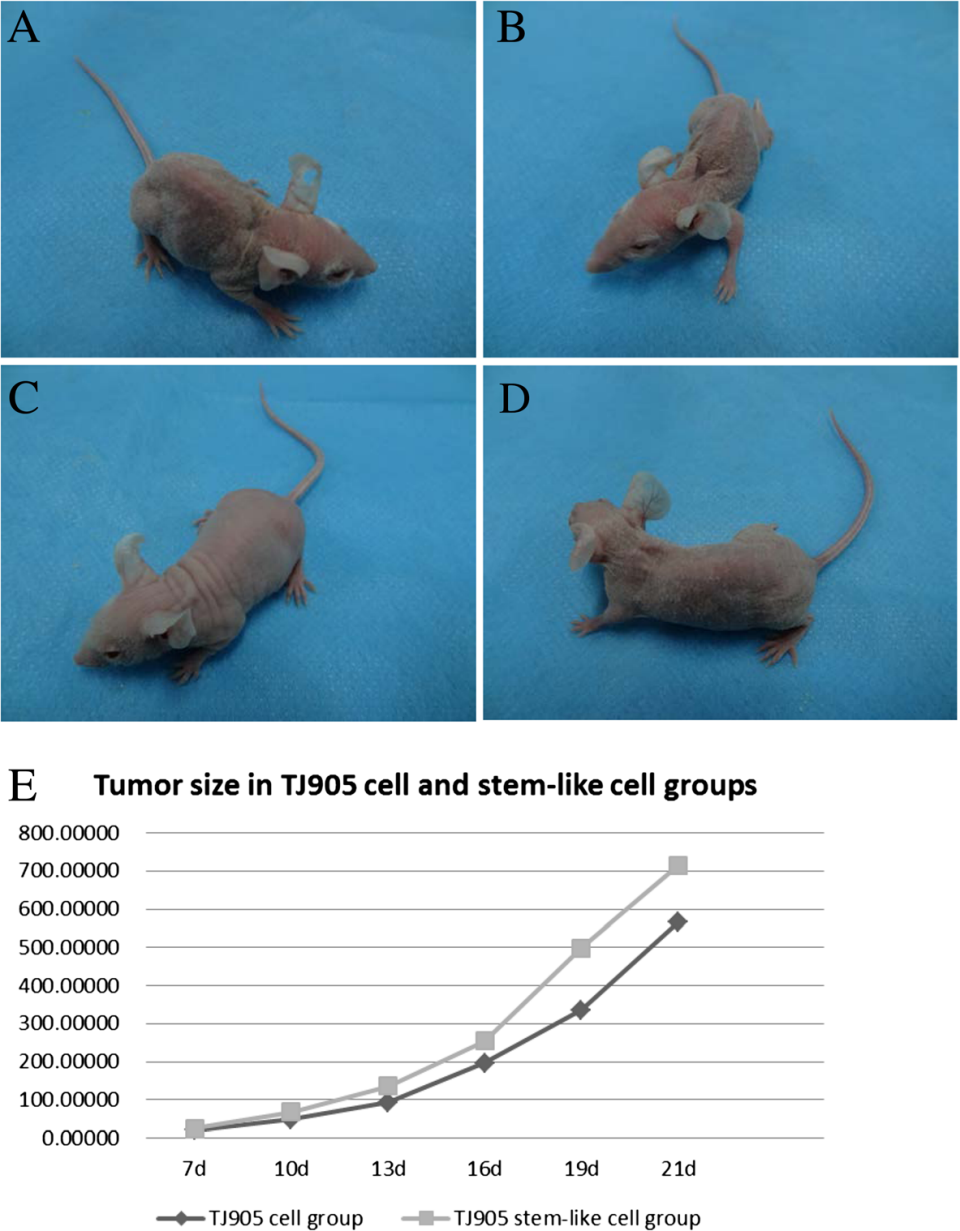
Observation on mice appearance. The pictures were shot at 14th post-injection day. **a** The mice in TJ905 cell group showed tarnished skin with depression; **b** The mice in TJ905 stem cell group showed similar manifestation to that in TJ905 cell group and additionally skin gradually became dark gray and weight loss occurred; **c** The mice in normal control group showed lustrous skin; **d** The mice in TJ905 CD133- cell group showed similar manifestation to that in TJ905 cell group; **e** The tumor size in stem-like cell group showed significantly larger than in TJ905 cell group

In TJ905 cell group, at 2 days after inoculation the appetite and foraging ability of mice increased and weight gradually rose. At 13–17 days after inoculation the mice started to show depressed with reduced activity, tarnished skin, and a little decline of foraging and eating ability. At 17 days after inoculation the symptom significantly aggravated. In TJ905 stem cell group, at 2 days after inoculation the living condition was similar to that in TJ905 cell group. At 10–12 days after inoculation, the mice showed similar manifestation to that in TJ905 cell group and additionally skin gradually became dark gray and weight loss occurred. At 13 days after inoculation the symptom significantly worsened with cachexia. In TJ905CD133- group, the mice growing condition and behavior were similar to that in TJ905 cell group. In normal control group, 2 days after inoculation, the mice returned to normal eating and drinking with obvious weight gain. They survived well without symptom and death (Fig. 1a-d). The curves of tumor growth were described in Fig. 1e.

### Tumor growth

The sorting rate of CD133+ cell was 0.64–0.91 %. Both tumor forming rate of TJ905 cell and stem cell group was 100 %. The tumor growth was infiltrative with round or oval shape and envelope could be observed, and the tumor showed darker color than the normal tissue around. The facet of tumor was fish-like with hemorrhage and necrosis. When the tumor grew big enough, the margin of tumor in TJ905 cell group and TJ905 CD133- group were clear, while in TJ905 stem cell group the boundary was significantly less clear. All in TJ905 cell, TJ905 CD133- and stem cell group there was no metastasis in peritoneal cavity. Normal control group showed no tumor formation.

### Tumor size

At 7, 10, 13, 16, 19, 21 days after inoculation comparison of tumor size between TJ905 cell, and stem cell group was significantly different (*P* <0.05). The tumor size in TJ905 stem cell group was much larger.

### Pathological morphology

**Fig. 2:**
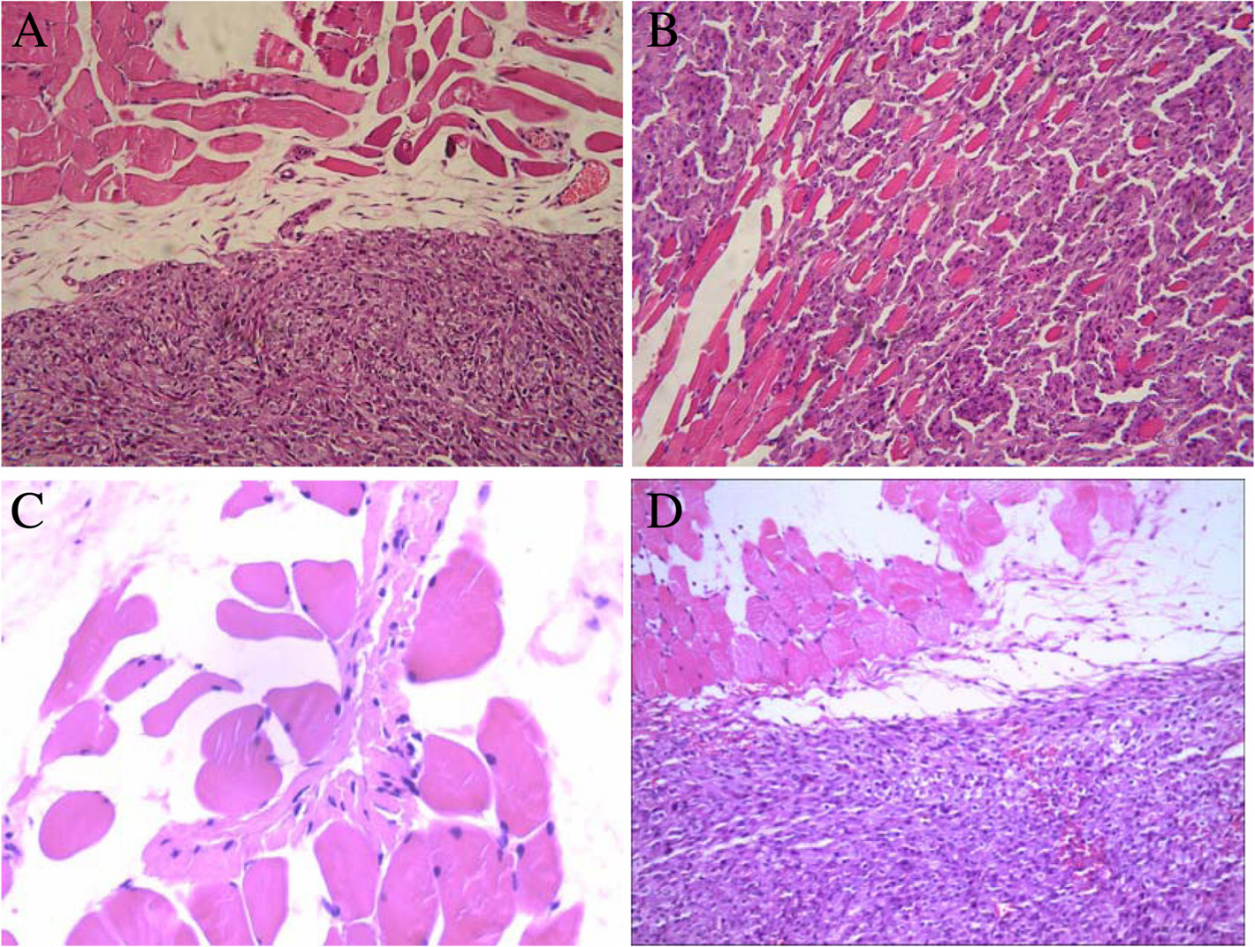
Observation on pathological morphology (×20). **a** In TJ905 cell group there was obvious boundary between normal tissue and tumor; **b** In TJ905 stem cell group the boundary between normal tissue and tumor was blurred; the cells showed pseudo-barrier-like or coral-like arrangement and dense clusters with necrosis, infiltrating into normal tissue; **c** In normal control group, skeletomuscular tissue was presented; **d** The mice in TJ905 CD133- cell group showed similar manifestation to that in TJ905 cell group

In TJ905 cell group, after HE staining the transplanted tumor showed round or spindle-shape with dense arrangement under light microscope, accompanied with big nucleolus, inversion of nucleus-cytoplasm rate, obvious nuclear atypia. Pathological caryokinesis, low-degree differentiation implied high degree in malignancy. Although tumor cell growth was infiltrative, there was still an obvious boundary between tumor and normal tissue. The pathological features in TJ905 CD133- group were similar to that in TJ905 cell group. In TJ905 stem cell group, the cells showed pseudo-barrier-like or coral-like arrangement and dense clusters, infiltrating into normal subcutaneous tissue. Infiltration of tumor cell could be seen at the boundary between tumor and normal tissue, and the boundary was blurred. Cystolization, necrosis and formation of neonatal vessel in the tumor center showed stronger malignant transformation. In normal control group, the staining showed skeletomuscular cells (Fig. 2).

### GFAP staining

**Fig. 3:**
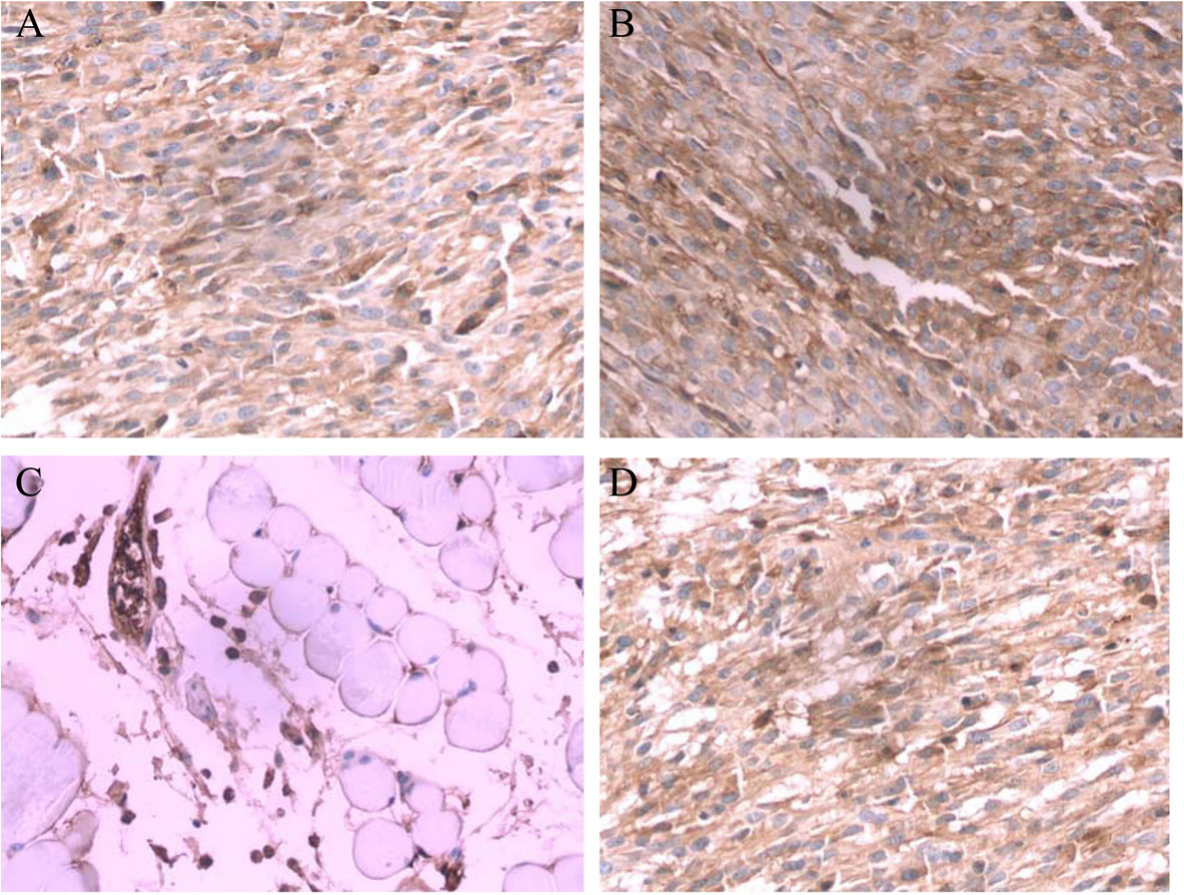
GFAP staining (×40). **a** The positive cells of GFAP staining in TJ905 cell group presented brown; **b** The positive cells of GFAP staining in TJ905 stem cell group also showed brown but were more than the cells in TJ905 cell group; **c** Skeletomuscular tissue was shown in normal control group; **d** The mice in TJ905 CD133- cell group showed similar manifestation to that in TJ905 cell group

GFAP staining in TJ905 cell, TJ905 CD133- and stem cell group were positive, showing buffy or brown. The positive rate in TJ905 stem cell group was significantly higher than that in TJ905 cell and CD133- group (*P* < 0.05) (Fig. 3).

### Ki-67 staining

**Fig. 4:**
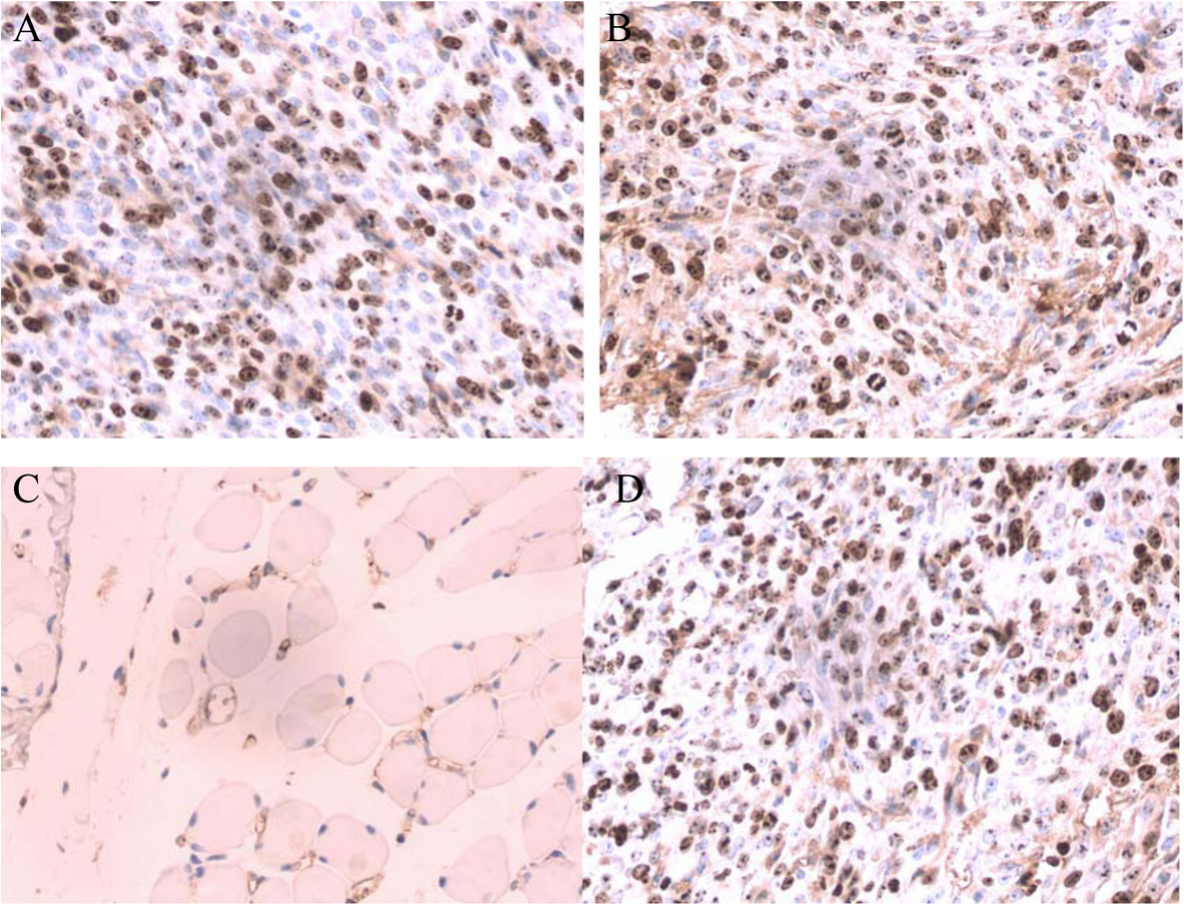
Ki-67 staining (×40). **a** The positive cells of Ki-67 staining in TJ905 cell group presented brown; **b** The positive cells of Ki-67 staining in TJ905 stem cell group were significantly more than that in TJ905 cell group; **c** The positive cells of Ki-67 staining in normal control group were rare; **d** The mice in TJ905 CD133- cell group showed similar manifestation to that in TJ905 cell group

KI-67 is a nuclear protein that is associated with cellular proliferation. Ki-67 is an excellent marker to determine the growth fraction of tumor cells, which is often correlated with the clinical course of cancer, especially the carcinomas of brain. Ki-67 staining in TJ905 cell, TJ905 CD133- and stem cell group were positive, showing buffy or brown. The positive rate in TJ905 stem cell group was significantly higher than that in TJ905 cell and CD133- group (*P* < 0.05) (Fig. 4).

### Olig-2 staining

**Fig. 5:**
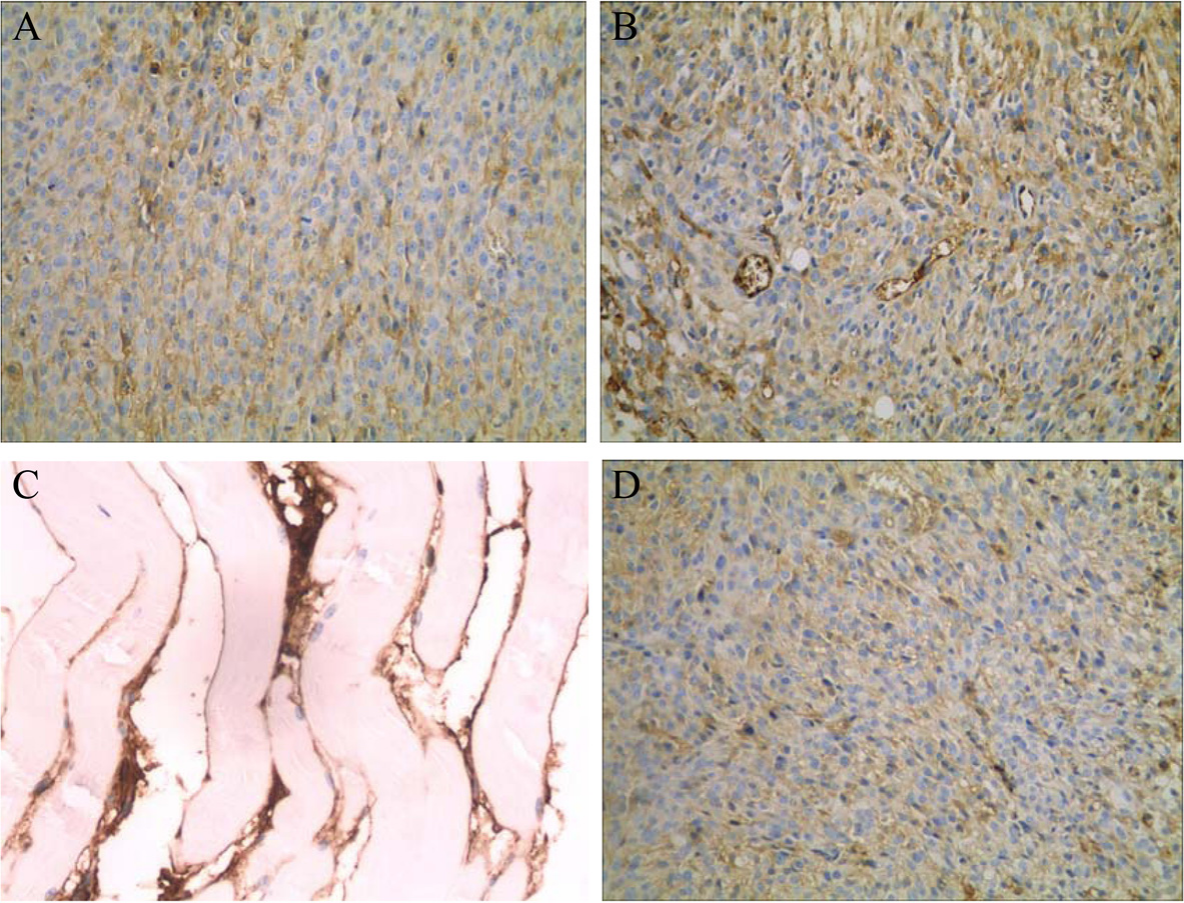
Olig-2 staining (×40). **a** The positive cells of Olig-2 staining in TJ905 cell group presented brown; **b** The positive cells of Olig-2 staining in TJ905 stem cell group were significantly more than that in TJ905 cell group; **c** The positive cells of Olig-2 staining in normal control group were rare; **d** The mice in TJ905 CD133- cell group showed similar manifestation to that in TJ905 cell group

Olig-2 is universally expressed in diffuse gliomas and serves as a diagnostic marker for brain tumor, especially for highly tumorigenic gliomas. Olig-2 staining in TJ905 cell, TJ905 CD133- and stem cell group were positive, showing buffy or brown. The positive rate in TJ905 stem cell group was significantly higher than that in TJ905 cell and CD133- group (*P* < 0.05) (Fig. 5).

### CD34 staining

**Fig. 6:**
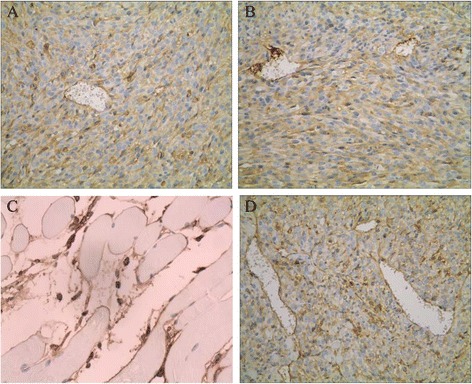
CD34 staining (×40). **a** The positive cells of CD34 staining in TJ905 cell group presented brown; **b** The positive cells of CD34 staining in TJ905 stem cell group were significantly more than that in TJ905 cell group; **c** The positive cells of CD34 staining in normal control group were notably less than that in the other two groups; **d** The mice in TJ905 CD133- cell group showed similar manifestation to that in TJ905 cell group

CD34 expresses in vascular endothelial cells, tumor cytoplasm or membrane. Average of MVP in TJ905 stem cells group was significantly higher than that in TJ905 cell and CD133- group (*P* < 0.05). Most capillaries of tumor tissue and cytoplasm structure were abnormal, and the basement membrane was incomplete or even constituted by only a single cell or only by blood tunnel. Some “blood vessels” were constituted by tumor cells and endothelium (Fig. 6).

## Discussion

Malignant gliomas are the most common primary neuroectodermal tumor, accounting for 15–23 % intracranial tumors in adults and about 50 % of all primary brain tumors. Despite technological progress in surgery, chemotherapy and radiotherapy, the rapid growth of tumor, invasive proliferation, and angiogenesis with high density make glioblastoma multiforme poor prognosis [12]. Although the specific mechanisms of cancer stem-like cells are still unclear, the theory will help us understand the formation of glioma. It can be postulated that the cancer stem-like cells will become a potential new target for cancer treatment [13].

Although various malignant glioma models have been established, the models created by traditional xenograft cell lines show limited invasiveness and heterogeneity, and changes of pathological characteristics for human gliomas. Recently, cancer stem-like cells isolated from surgical specimens were used to set up new animal model, which showed more similar characteristics of human cancer [14]. Therefore, it is necessary to explore the model established by glioma stem cells in order to study characteristics of glioma and provide an ideal platform to address the mechanisms of tumor recurrence and chemoresistance.

It was reported that tumor could be formed when NOD-SCID nude mice was injected 100 CD133+ cells extracted from cancer tissue, but injection with 100,000 CD133- cells did not form tumors. That heterogeneity showed only a small part of cancer cells might be a key for tumor recurrence, and targeted treatment of those cells might be new innovative method to eradicate the tumor [15]. We used CD133+ TJ905 stem cells injected subcutaneously in nude mice to form glioma model, which provided a vector for studying the characteristics of malignant glioma.

In this study, we established glioma model by using TJ905 cells and stem cells, and all of experimental animals formed tumor. Compared with model of TJ905 cells, the model of glioma stem cells showed great differences in growth and behavior condition, such as weight loss, activity, skin, foraging and eating ability, cachexia, survival rate and exudate. The tumor weight, growth time and pathological examination also matched these findings.

GFAP, an intermediate fibrous protein, is a marker of astrocyte and a symbol of differentiation into mature cells from brain tumor stem cells [16]. In our study, both of the tumor models by TJ905 cells and stem cells highly expressed GFAP, indicating that the tumor originated from astrocytes or differentiated into mature cells. The Ki-67 index is a marker of proliferation in malignant tumors, especially brain cancers, as it can be detected during all the active phases of cell cycle but is absent in resting cells [17]. It is commonly used as a complement to grading systems that include mitotic counting as a sign of proliferation [18]. Olig-2 is well recognized as a diagnostic marker for brain tumor, for it is required for proliferation of human glioma cells [19]. In our research, significant expression of Ki-67 and Olig-2 was observed in TJ905 cells and was much higher in TJ905 stem cells, which demonstrated that the malignant degree in cancer stem-like cells.

CD34 is a salivary mucin on cell surface and widely used to purify hematopoietic stem cells and as a marker of most capillary endothelial cells. Angiogenesis is not only an important process involved in the normal growth and differentiation, but necessary for development of a tumor. The higher the degree of malignancy is, with the faster the growth, the more adequate supply of oxygen and nutrients are needed. MVD (microvessel density) of tumor may indirectly reflect growth, invasion and metastasis of tumor tissue [20]. Thereby MVD is used to determine the grade and prognosis of malignant tumor. In our study, in TJ905 stem cell group the CD34 expression was much higher than that in TJ905 cell group, which also shows the transplanted tumor that originates from cancer stem-like cell has a stronger malignance.

In summary, the tumor model from CD133+ GBM stem-like cells showed the features from astrocytes with high malignance. Based on our previous researches [21, 22], we established xenograft tumor model by using TJ905 cells and stem cells, and found the animal model from glioma stem cells might exhibit more malignant characteristics in the detected markers. We would further explore the molecular mechanisms of this GBM animal model in the future in order to find a platform for faithfully mirroring clinical practice.

## References

[1] Nieto-Sampedro M, Valle-Argos B, Gómez-Nicola D, Fernández-Mayoralas A, Nieto-Díaz M (2011). Inhibitors of glioma growth that reveal the tumour to the immune system. Clin Med Insights Oncol.

[2] Tafani M, Di Vito M, Frati A, Pellegrini L, De Santis E, Sette G (2011). Pro-inflammatory gene expression in solid glioblastoma microenvironment and in hypoxic stem cells from human glioblastoma. J Neuroinflammation.

[3] Anton K, Baehring JM, Mayer T (2012). Glioblastoma multiforme: overview of current treatment and future perspectives. Hematol Oncol Clin North Am.

[4] Qiu H1, Fang X, Luo Q, Ouyang G. Cancer stem cells: a potential target for cancer therapy. Cell Mol Life Sci. 2015. [Epub ahead of print] doi:10.1007/s00018-015-1920-4. 10.1007/s00018-015-1920-4 PMC11113644 25967289

[5] Cho DY, Lin SZ, Yang WK, Lee HC, Hsu DM, Lin HL (2013). Targeting cancer stem cells for treatment of glioblastoma multiforme. Cell Transplant.

[6] Choy W, Nagasawa DT, Trang A, Thill K, Spasic M, Yang I (2012). CD133 as a marker for regulation and potential for targeted therapies in glioblastoma multiforme. Neurosurg Clin N Am.

[7] Ernst A, Hofmann S, Ahmadi R, Becker N, Korshunov A, Engel F (2009). Genomic and expression profiling of glioblastoma stem cell-like spheroid cultures identifies novel tumor-relevant genes associated with survival. Clin Cancer Res.

[8] Jin F, Gao C, Zhao L, Zhang H, Wang HT, Shao T (2011). Using CD133 positive U251 glioblastoma stem cells to establish nude mice model of transplanted tumor. Brain Res.

[9] Zhang A, Hao J, Wang K, Huang Q, Yu K, Kang C (2013). Down-regulation of miR-106b suppresses the growth of human glioma cells. J Neurooncol.

[10] Jin F, Zhao L, Guo YJ, Zhao WJ, Zhang H, Wang HT (2010). Influence of Etoposide on anti-apoptotic and multidrug resistance-associated protein genes in CD133 positive U251 glioblastoma stem-like cells. Brain Res.

[11] Jin F, Cheng D, Tao JY, Zhang SL, Pang R, Guo YJ (2013). Anti-inflammatory and anti-oxidative effects of corilagin in a rat model of acute cholestasis. BMC Gastroenterol.

[12] Ahmadloo N, Kani AA, Mohammadianpanah M, Nasrolahi H, Omidvari S, Mosalaei A (2013). Treatment outcome and prognostic factors of adult glioblastoma multiforme. J Egypt Natl Canc Inst.

[13] Zhang X, Zhang W, Mao XG, Zhen HN, Cao WD, Hu SJ (2013). Targeting role of glioma stem cells for glioblastoma multiforme. Curr Med Chem.

[14] Sampetrean O, Saga I, Nakanishi M, Sugihara E, Fukaya R, Onishi N (2011). Invasion precedes tumor mass formation in a malignant brain tumor model of genetically modified neural stem cells. Neoplasia.

[15] Tunici P, Irvin D, Liu G, Yuan X, Zhaohui Z, Ng H (2006). Brain tumor stem cells: new targets for clinical treatments?. Neurosurg Focus.

[16] Yeo S, Bandyopadhyay S, Messing A, Brenner M (2013). Transgenic analysis of GFAP promoter elements. Glia.

[17] Jakobsen JN, Sørensen JB (2013). Clinical impact of ki-67 labeling index in non-small cell lung cancer. Lung Cancer.

[18] Luporsi E, André F, Spyratos F, Martin PM, Jacquemier J, Penault-Llorca F (2012). Ki-67: level of evidence and methodological considerations for its role in the clinical management of breast cancer: analytical and critical review. Breast Cancer Res Treat.

[19] Dougherty JD, Fomchenko EI, Akuffo AA, Schmidt E, Helmy KY, Bazzoli E (2012). Candidate pathways for promoting differentiation or quiescence of oligodendrocyte progenitor-like cells in glioma. Cancer Res.

[20] Sica G, Lama G, Anile C, Geloso MC, La Torre G, De Bonis P (2011). Assessment of angiogenesis by CD105 and nestin expression in peritumor tissue of glioblastoma. Int J Oncol.

[21] Jin F, Zhao L, Zhao HY, Guo SG, Feng J, Jiang XB (2008). Comparison between cells and cancer stem-like cells isolated from glioblastoma and astrocytoma on expression of anti-apoptotic and multidrug resistance-associated protein genes. Neuroscience.

[22] Jin F, Zhao L, Zhao HY, Guo SG, Feng J, Jiang XB (2008). Paradoxical expression of anti-apoptotic and MRP genes on cancer stem-like cell isolated from TJ905 glioblastoma multiforme cell line. Cancer Invest.

